# Discovery of chemically diverse compounds targeting the *Plasmodium falciparum* coenzyme A pathway

**DOI:** 10.1186/1475-2875-13-S1-P34

**Published:** 2014-09-22

**Authors:** Sabine Fletcher, Vicky Avery

**Affiliations:** 1 Griffith University, Brisbane, QLD, Australia

## Background

Drug resistance has rendered previous generation anti-malarials ineffective and is also rapidly emerging against the current therapeutics of choice, artemisinin and its derivatives, making the discovery of new anti-malarials with novel mechanisms of action a priority. The Coenzyme A (CoA) synthesis pathway is a well-known anti-microbial drug target that is also essential for the malaria parasite *Plasmodium falciparum*. To date, this pathway has not been exploited in anti-malarial drug development. Herein a novel high throughput approach for the identification of chemically diverse inhibitors of the CoA synthesis pathway from chemical compound libraries is reported.

## Materials and methods

To identify novel CoA synthesis pathway inhibitors, a chemical rescue screening approach was developed by modifying a well-established *P. falciparum* imaging assay. In short, a test compound was considered likely to inhibit the *P. falciparum* CoA synthesis pathway if addition of the pathway’s end product, CoA, was able to negate the compound’s growth-inhibitory action on *P. falciparum* asexual parasites.

## Results

The chemical rescue approach was employed to screen the Medicines for Malaria Venture (MMV) malaria box and another small focussed compound library. This resulted in the identification of 12 chemically diverse inhibitors. To ascertain accurate potency and selectivity, the half maximal inhibitory concentration (IC_50_) of these compounds was determined for both *P. falciparum* and human embryonic kidney cells. Seven compounds showed sub-micromolar activity against the parasite, with selectivity indices ranging between 6 and over 300 times. CoA supplementation was confirmed to alleviate the effects on parasite growth and cell viability in a dose dependant manner. Microscopic investigation of the stage of effect and phenotype of treated parasites was performed on a selection of the active compounds.

## Conclusions

Investigation of two medium-sized prioritised compound libraries resulted in the identification of a set of chemically diverse CoA synthesis pathway inhibitors with IC_50_ values ranging between 120 nM and 6 μM. The chemical rescue approach for the CoA pathway yielded hit compounds with demonstrated anti-plasmodial action and defined species selectivity. An additional benefit of such an approach is the first step in the determination of the mode of action of novel anti-malarial compounds, namely inhibition of the CoA synthesis pathway. Collectively, we propose the CoA rescue approach as a valuable novel tool for future anti-malarial drug discovery.

